# Lactose and Galactose Content in Spanish Cheeses: Usefulness in the Dietary Treatment of Patients with Galactosaemia

**DOI:** 10.3390/nu15030594

**Published:** 2023-01-23

**Authors:** Isidro Vitoria, Fuensanta Melendreras, Antonio Vázquez-Palazón, Dolores Rausell, Patricia Correcher, Domingo González-Lamuño, Mónica García-Peris

**Affiliations:** 1 Nutrition and Metabolopathies Unit, La Fe University Hospital, 46025 Valencia, Spain; 2 National Technological Center for Food and Canning Industries, 30500 Murcia, Spain; 3 Metabolopathies Laboratory, La Fe University Hospital, 46026 Valencia, Spain; 4 Pediatric Nephrology and Metabolism, “Marqués de Valdecilla” University Hospital, 39008 Santander, Spain

**Keywords:** inborn errors of metabolism, galactosaemia, galactose restriction, cheese, lactose, galactose

## Abstract

In galactosaemia, a strict galactose-free diet is necessary to prevent or resolve acute symptoms in infants. However, because the body produces up to 10 times more galactose than is found in a galactose-restricted diet, excessively restrictive diets should be avoided in children and adults to prevent nutritional deficiencies. Since cheese is a nutritional source of the calcium necessary for bone health, the latest international guidelines on the management of classical galactosaemia (2017) allow the consumption of cured cheeses with less than 25 mg of galactose/100 g and recommend that each country verifies the adequacy of the cheeses, since most mature cheeses do not always have a lower galactose content. In total, 32 cheese samples were purchased (19 Spanish and 13 international cheeses), and their lactose and galactose contents were analysed using ion chromatography with pulsed amperometric detection (IC-PAD). Five Spanish cheeses contained less than 25 mg of galactose/100 g: García Baquero semi-cured cheese; Hacendado, Gran Reserva and Mahón cured cheeses; and García Baquero Reserva 12-month cured cheese. In addition, eight international cheeses were confirmed as suitable: Comté, Gouda, Gruyere, Maasdam, Parmigiano, Edam, Emmental, and some samples of Cheddar. In addition to the well-known low-galactose Swiss and Dutch cheeses, according to the current results, five Spanish cheeses can be safely consumed. The greater availability of types of cheese favours better bone health in patients with galactosaemia.

## 1. Introduction

Classic galactosaemia (MIM 230400) is an inborn error of the metabolism caused by a deficiency in the enzyme galactose-1-phosphate uridyl transferase, which leads to the accumulation of galactose metabolites and causes severe liver disease, sepsis, and cataracts in infants, and long-term complications such as cognitive and ovarian dysfunction [1]. Its incidence is 1:19,000 to 1:44,000 in Europe and the United States, and it is more common in Ireland [2]. Galactosaemia due to galactokinase deficiency (MIM 230200) is associated with cataracts, and galactosaemia caused by epimerase deficiency (MIM 230350) is rarer. In all three cases, treatment consists of a lifelong low-galactose diet. The main source of galactose is the disaccharide lactose, which is present in milk, dairy products, and processed foods containing milk.

In recent years, the possibility of offering foods containing low amounts of galactose, such as certain cheeses, has been raised, with the aim of increasing bone mineralisation through calcium intake. This change in recommendation is based on the idea that endogenous galactose synthesis is important and would probably be the cause of cognitive impairments, speech abnormalities, neurological abnormalities, behaviour abnormalities, and the very high prevalence of primary ovarian insufficiency in affected females [3]. Endogenous galactose production is estimated to be 580 mg/day for a 70 kg adult [4]. In addition, endogenous galactose production does not appear to be affected by exogenous galactose intake [5].

On the other hand, bone density in adults with galactosaemia is low [6]. The pathophysiologic mechanisms underlying skeletal losses in patients with classic galactosaemia are not well understood. However, several have been proposed, including nutritional deficiencies, primary ovarian insufficiency in women and intrinsic factors related to bone metabolism [7].

According to a survey of dietitians in 16 European countries in 2002 [8], it was accepted that some types of aged cheese contain little lactose and galactose, so that 12 dietitians (75%) allowed some types of aged and hard cheese, particularly Emmental, Gruyere, and Tilsiter. Similar recommendations were made in Bernstein’s (2022) classic book on nutrition in galactosaemia [9].

The latest international guidelines on the management of classic galactosaemia in 2017 [10] allow the consumption of mature cheeses containing less than 25 mg galactose/100 g. In this regard, the consumption of old and aged cheeses such as Jarlsberg, Emmental, Swiss, Gruyere, Tilsiter, mature Parmesan, and mature Cheddar cheese is allowed. They also recommend that this list be adapted to the particular characteristics of each country. Thus, the UK Galactosaemia Association provides information about the cheeses permitted in the UK [11] and added three more cheeses in 2022: low-fat UK extra mature Cheddar cheese, Pecorino Romano, and Babybel—Original.

The inclusion of new cheeses specific to a country is an interesting measure to improve the bone health of patients with galactosaemia. The aim of this study was to analyse the lactose and galactose content of cheeses consumed in Spain, both produced in our country and those of international origin.

## 2. Materials and Methods

In the months of July to October 2022, 32 cheese samples were purchased directly from a shopping centre (supermarket or hypermarket).

The samples were prepared by homogenisation. After grinding the product, 0.5 g of the sample was weighed in 50 mL centrifuge tubes on an analytical balance with a precision of 0.1 mg, and the sample was made up to 50 mL with ultrapure water (Type 1) then heated to 50–60 °C. The samples were then stirred for 30 min in a waterwheel at 60 rpm and 45 min in a centrifuge at 4600 rpm.

The measurements were carried out in triplicate with a Metrohm ion chromatograph (model 930 Compact IC Flex) with a pulse amperometric detector. A Metrosep Carb2 separation column was used. After microfiltration with membrane filters (0.45 µm and 0.22 µm), the samples were injected. In addition, the equipment had a 0.2 µm in-line ultrafiltration system incorporated in the autosampler.

For the quantitative determination of lactose and galactose, 2 different straight lines were used, with ranges between 0.02 and 0.5 mg/100 g, and between 0.2 and 2.5 mg/100 g. The first straight line was used for quantification of the lower concentrations, while the second one was used for quantification of the higher concentrations. Since the starting dilution of the samples was 1:10 and the lowest concentration point was discarded, the quantification of samples with the lower line was from 0.5 to 5.0 mg/100 g, and that with the high line was from 5.0 to 25.0 mg/100 g. For the quantification of samples with values above 25.0 mg/100 g, additional dilutions were applied to the sample in order to quantify them with the high-concentration line.

The total duration of the chromatogram was 40 min, and it could be seen that galactose was eluted at Minute 10 and lactose at approximately Minute 15.5, depending on the properties of the mobile phase used. To ensure the results were based on ISO 17025 quality standards, the sequence was complemented with verification standards and fortified samples. These controls met the acceptance and rejection criteria for sequential quality control and were in accordance with the validation of the test method, both for replicate samples, fortified samples, and reference materials.

## 3. Results

The following Table 1 shows the different cheeses analysed, stating the supermarket or hypermarket of purchase, the country of origin, the type of cheese, the source of the milk (goat, sheep, cow, or mixed), the manufacturer, the brand, the batch, and the expiry date.

Alongside the traceability information, the results obtained on the lactose and galactose contents are described in Table 2. The units are expressed in mg/100 g of food, with a value of 5 mg/100 g being the limit of quantification. The galactose equivalent is calculated from by the sum of the galactose concentration and half of the lactose concentration (from a lactose molecule, a glucose and a galactose molecule are formed through hydrolysis of the intestinal lactase). When the values to be added include a detection limit (<5 mg/100 g), the most unfavourable value has been applied to obtain a theoretical maximum value of galactose.

## 4. Discussion

Cheeses are classified according to their rheological properties as very hard, hard, semi-hard, and soft [12]. Depending on their ripening time, they are classified as soft (less than 30 days), semi-cured (one to three months), cured (three to six months), mature (six to nine months), and aged (more than nine months).

The current study included samples of cheeses that patients can easily find in supermarkets and avoided artisan cheeses or cheeses from cheese dairies with a more limited distribution. Therefore, out of the 19 Spanish cheeses analysed, there were only 5 with a DOP label; in Spain, 26 different types of DOP cheeses are recognised.

In the present study, the first striking finding is that, of the 32 cheeses analysed, only 2 (Aged Hacendado and Punteiro Tetilla DOP) contained more than 5 mg lactose/100 g. Thus, the galactose content should be the limiting factor for recommending a particular brand of cheese for patients with galactosaemia.

In order to compare the lactose and galactose content of the 32 different types of cheeses analysed, they were grouped into 5 types of cheese: soft cheeses of Spanish origin (1 brand), semi-cured cheeses of Spanish origin (5 brands), cured cheeses of Spanish origin (8 brands), mature and aged cheeses of Spanish origin (5 brands), and cheeses of international origin (13 brands).

The only example of soft cheese of Spanish origin (Tetilla DOP) had cow’s milk as its source. It contained 11.8 mg of lactose and 13.9 mg of galactose per 100 g. The equivalent galactose content was 19.8 mg/100 g, which, despite being a high value, was a lower value than expected for this group of cheeses.

The five brands of semi-cured cheeses of Spanish origin (Table 3) had a mixture of cow, goat, and sheep milk as their source. All of them contained less than 5 mg/100 g lactose. The semi-cured García Baquero cheese could be of interest to patients with galactosaemia, as it also contains < 6.1 mg galactose/100 g, giving an equivalent galactose content of <8.6 mg/100 g. The other four cheeses contained 32.7 to 115.3 mg/100 g galactose.

In relation to the group of eight semi-cured cheeses of Spanish origin (Table 4), there were three cheeses with less than 25 mg/100 g galactose equivalent. These were two cheeses made from a mixture of cow, sheep, and goat milk, namely Cured Hacendado and Gran Reserva Cured Lidl, and one cow’s milk cheese from Menorca (Cured Mahón DOP). The other five cheeses included in this group contained less than 5 mg/100 g lactose, but from 33.3 to 559.1 mg/100 g galactose.

Of the five mature, old, and aged Spanish cheeses, only one (García Baquero, with a mixed milk origin) contained less than 14.8 mg galactose equivalent/100 g (Table 5). The other four contained from <44 to <324.9 mg galactose equivalent/100 g. Of these, three were from sheep’s milk (Mature Flor de Esgueva, Mature Hacendado and Aged Sheep’s Manchego) and one from a mixture of milks (Mature García Baquero).

In relation to the cheeses of international origin, all cheeses with a maturity of more than nine months made from cow’s milk, except for Cheddar cheese, contained from <7.5 to <11.1 mg/100 g of galactose equivalent (Table 6).

Thus, the Comté cheese aged for 12 months contained <5 mg lactose/100 g and 8.6 mg galactose/100 g. In another study [13], a Comté mild French mountain cheese contained <0.05 mg lactose and up to 1.86 mg galactose per 100 g.

The two Gouda cheeses analysed (Old Amsterdam Cured Gouda and Aged Gouda) contained <8 mg galactose equivalent/100 g. Previous studies [14] indicated that some Gouda cheeses had less than 20 mg lactose/100 g (Deli Asda Gouda, Finest Mature Dutch Gouda, Sainsbury’s Gouda, and Waitrose Gouda) but others had 30 to 140 mg lactose/100 g (Tesco Continental, Sainsbury’s Dutch Gouda, Sainsbury’s Mature Gouda, Asda Gouda).

The two types of Gruyere cheese analysed contained less than 7.5 mg galactose equivalent per 100 g, as did the cheeses reported in the literature, all with <2.8 to <10 mg lactose/100 g and < 0.05 to 11.78 mg galactose/100 g (Safeway Swiss, Sainsbury’s Swiss, Tesco Swiss Gruyere Block, Crystal Farms, Whole Foods Market, and Brennan’s Cellars) [14,15].

In relation to the Cheddar cheeses analysed, one contained up to 285.5 mg galactose/100 g while the other cheese, which was a cured cheese of the same brand, contained less than 7.5 mg galactose equivalent/100 g. Very different concentrations were also reported in the literature. Thus, while samples of Valley Spire West Country, Parkham, Lye Cross Vintage, Lye Cross Mature, Tesco West Country Farmhouse Extra Mature, and Sainsbury’s and TTD West Country Farmhouse Extra Mature cheeses had average lactose and galactose levels less than 15 mg/100 g (range: <0.05 to 12.65 mg) [13], other studies [14,15] reported cheeses with <2.8 to 390 mg galactose per 100 g (Old Cheddar Cracker Barrel, Cheddar Kraft, Safeway Mild Welsh Cheddar). A study of cheeses from Ireland also shows a similar disparity in the results for Cheddar cheeses, with a galactose content between 32 and 908 mg/100 g [16].

The Parmesan cheeses analysed (Deluxe Parmigiano and Cured Grana Padano) contained less than 7.5 mg galactose equivalent/100 g. Van Calcar’s study [15] reported contents of 23.6 (powdered Kraft) and < 5.1 mg galactose/100 g (Brennan’s Cellars), while in Portnoi’s study [14], most of the cheeses analysed (Sainsbury’s Parmiggiano Reggiano, Tesco Finest Parmesan, House of Fraser Parmesan, Ferrari Tasso Grana Padano, and Tesco Italian Grana Padano) contained less than 2.8 mg lactose/100 g, while only Tesco Fresh Italian Parmesan contained 30 mg lactose/100 g and <10 mg galactose/100 g. Monti et al. found a concentration of 0.27 mg galactose/100 g in Grana Padano cheese [17].

The mature Edam cheese tested contained less than 7.5 mg galactose equivalent/100 g. Portnoi’s 2009 study [14] reported two brands with less than 20 mg lactose and 10 mg galactose/100 g (Asda Edam Wedge and Waitrose Edam), but four others with 97 to 294 mg lactose/100 g (Sainsbury’s Dutch Edam, Frico Organic Edam, Morrisons Dutch Edam, and Ada Dutch Edam Wedge). In 2011, Portnoi [18] reported that the original Mini Babybel cheese (with Edam cheese) contained <2 to 11.7 mg lactose per 100 g.

The Emmental cheese analysed (mature Milbona Emmental) contained less than 7.5 mg/100 g galactose equivalent, equivalent to values of <0.05–10 mg lactose/100 g and 2.19 to <5.1 mg galactose/100 g found in previous studies, e.g., Safeway Swiss Emmental, President Emmental, President Sliced Emmental, President Grated Emmental, and Sainsbury’s Swiss [14]; Whole Foods Market and Breman’s Cellar) [15]; Mini BabyBel Emmental [18] and Emmi Swiss Fondue [13].

Finally, the other two international cheeses studied (mature Maasdam and Havarti) also contained less than 7.5 mg galactose equivalent/100 g.

For the production of cheese, it is important to form a curd, which is facilitated by casein, which is present in mammalian milk. The carbohydrate fermentation profiles differ among the treatments during cheese making and ripening. Briefly, in cheese production, there are two factors that reduce the lactose content. Firstly, the separation and subsequent removal of the whey (curdling or coagulation of the milk), and the lactic acid bacteria containing lactase release glucose and galactose can be introduced. Glucose can be used as an energy source and some lactic acid bacteria can also convert galactose into glucose.

Some cheeses add a starter culture of bacteria that increases the lactic acid concentrations and thus reduces the lactose concentration. It is generally accepted that the longer the cheese matures, the lower the lactose content will be. However, according to analyses of mature and aged Spanish cheeses, we were not always able to verify this. However, most of the mature or aged cheeses from abroad contain hardly any lactose or galactose. What they have in common is that they are made from cow’s milk. However, there are also exceptions in Spanish cheeses; there are cheeses without galactose or lactose, whether from sheep’s milk, cow’s milk, or a mixture of milks [12].

The lactose and galactose content of cheeses Is probably influenced by several factors: the ripening time, the type of bacterial cultures used in the initial and subsequent culture, the steps of separating the curd from the lactose-rich whey, the processing temperature, the way the ripening is carried out (industrial or artisan methods), the season of the year, and the origin of the milk [19,20]. This study had the limitation that there may be some variation in the galactose content if commercial brands change the types of bacterial cultures used to make their cheeses, especially in the case of bacterial starter cultures [21]. As indicated by MacDonald (2009) [14], cheese is a biological medium and so all parameters will vary slightly. Many factors determine the final lactose and galactose content, and cheeses permitted in the case of galactosaemia should have repeated analyses over time to reconfirm the absence of significant amounts of galactose.

The dietary guidelines for galactosaemic patients provide advice on what to eat and drink to meet nutrient needs, promote health, and prevent complications. Among the most vexing decisions in the management of metabolic diseases that require the restriction of dietary components is the determination of what constitutes an adequate and sufficient diet. The most recent guidelines allow amounts of galactose greater than those previously considered safe [9,10].

Our paper provides data that should help patients to follow dietary recommendations to prevent certain complications associated with strict lactose–galactose-free diets. The study was performed with the Spanish Galactosemia Association to increase confidence in dietitians and families.

Since several cheeses are permitted for patients with galactosaemia, it is relevant to know the recommended daily amounts of these cheeses. According to the dietary guidelines for the Spanish population [22], two to three daily servings of dairy products are recommended throughout life, with two servings between the ages of two and eight years and three servings thereafter. These suggestions are similar to those of the American dietary guidelines [23,24], which recommend a daily intake of 2 to 2.5 cup equivalents per day for children aged two to three years, 2.5 cup equivalents per day for children aged four to eight years and 3 cups per day for children aged nine years and older. In general, one cup in the dairy group counts as one cup of milk or yoghurt, 1 1⁄2 ounce of natural cheese such as cheddar cheese or 2 ounces of soft cheese, i.e., one serving would be equivalent to 42 g of a mature or semi-cured cheese or 56 g of a fresh cheese. We must remember that patients with galactosaemia cannot consume other dairy products, such as milk or yoghurt.

The majority of international cheeses, as well as the Spanish cheeses included in this study, contain 180–300 mg of calcium per ounce (28 g) [25,26].

The daily intake of 1.5 servings (63 g) in children aged two to three years would mean an intake of 405–675 mg of calcium, i.e., 58–96 % of the recommended daily intake at these ages, which is 700 mg [27]. In four- to eight-year-old children, the intake of two servings (84 g) would provide 540–900 mg of calcium daily, which is 54–90 % of the recommended daily intake of 1000 mg of calcium. Similarly, in pre-adolescents and adolescents aged 9 to 18 years, a daily intake of 2.5 servings of cheese (105 g) would provide 675–1125 mg of calcium, which would be 52–86 % of the recommended daily requirement of 1300 mg of calcium at these ages. In adulthood, it would also probably be unnecessary to recommend more than 2.5 servings per day, as the daily requirement ranges from 1000 mg (in the population aged 19–50 years and men aged 51–70 years) to 1200 mg of calcium (in the population aged over 70 years and women aged 51–70 years), so the percentages of the daily calcium requirement met would range from 56 to 100%.

These recommendations for cheese consumption are indicative and should not be taken as a rigid rule. Not only would cheese intake help to improve the bone health of patients with galactosaemia due to its calcium content, but it also contains proteins of high biological value, potassium, phosphorus, magnesium, and vitamins D, B12, B1 (thiamine), and B2 (riboflavin) [28]. However, we are aware that patients with inborn errors of metabolism, such as galactosaemia, are very reluctant to incorporate foods that have been forbidden to them for so many years, so nutritional advice must be developed with prudence and personal tact.

## 5. Conclusions

In summary, apart from the well-known cheeses from other countries such as Emmental, Gruyere, Edam, Parmesan, and Gouda, according to the current results, there are a number of Spanish cheeses that patients with galactosaemia can consume because they have a galactose content of less than 25 mg/100 g, such as the semi-cured cheese García Baquero; the cured cheeses Hacendado, Gran Reserva, and Mahón; and the aged cheese García Baquero Reserva (12 months). The inclusion of more cheeses in the diet of patients with galactosaemia may allow a greater intake of calcium from natural foods, leading to better bone mineralisation, an aspect of great importance in this inborn error of metabolism.

## Figures and Tables

**Table 1:** Characteristics of the analysed cheeses (supermarket or hypermarket, country of origin, type, source of milk, manufacturer, brand, batch, and expiry date) (*) Blend: milk from cows, goats, and sheep in varying proportions. AOP/DOP: protected designation of origin.

Nº	Commercial Area	Country of Origin	Type	Source	Manufacturer	Brand Name	Lot	Expiry
1	Lidl	Spain	Semi-cured	Blend (*)	Quesos del Duero SA	Semi-cured Roncero	66220425 ZA 15	30/09/2022
2	Mercadona	Spain	Semi-cured	Blend (*)	Entrepinares	Semi-cured Hacendado	B63760	12/09/2022
3	Family M.	Spain	Semi-cured	Blend (*)	García Baquero	Semi-cured García Baquero	66220829	01/02/2023
4	Family M.	Spain	Semi-cured	Blend (*)	Lactalis	Semi-cured Gran Capitán	278A	24/09/2022
5	Family M.	Spain	Semi-cured	Blend (*)	Campofrío	Semi-cured Navidul	2422147	04/10/2022
6	Lidl	Spain	Cured	Cow and sheep	LACTALIS SL	Cured Roncero	247	14/08/2022
7	Mercadona	Spain	Cured	Blend (*)	Entrepinares	Cured Hacendado	B63520	05/09/2022
8	Family M	Spain	Cured	Sheep and cow	Lactalis	Cured Gran Capitán	269	02/02/2023
9	Lidl	Spain	Cured	Blend (*)	Quesos del Duero SA	Cured Gran Reserva	36210426	17/09/2022
10	Spar	Spain	Cured	Blend (*)	García Baquero	Cured García Baquero Maestría	66210518 ZR	14/09/2022
11	Lidl	Spain	Cured DOP	Goat	Quesería Montesinos	Cured Murcia al Vino DOP	21009-161 06	08/09/2022
12	Lidl	Spain	Cured DOP	Cow	Lacto Industrial Menorquina S.L.	Cured Mahón DOP	117	27/01/2023
13	Lidl	Spain	Cured DOP	Sheep	Quesos la Vasco-Navarra SA	Cured diazabal DOP	2422269	13/06/2023
14	Super Dumbo	Spain	Old	Blend (*)	García Baquero	García Baquero (10 months)	36210424 CR	03/12/2022
15	Family M	Spain	Old	Sheep	Ind. Láct. Peñafiel	Aged Flor de Esgueva	278AB	25/08/2022
16	Mercadona	Spain	Old	Sheep	Entrepinares	Aged Hacendado	B66227	24/11/2022
17	Family M	Spain	Reserva	Blend (*)	García Baquero	García Baquero Reserva (12 months)	36220211 ZA 50	16/02/2023
18	Lidl	Spain	AgedDOP	Sheep	Quesos Vega Sotuelamos SL	Aged Sheep’s Manchego DOP	232L3427D	23/02/2023
19	Mercadona	Spain	Soft DOP	Cow	Queizuar S.L.	Punteiro TetillaDOP	TM220779	15/03/2023
20	Lidl	France	Cured Comté DOP	Cow	Fromageries Vagne	Cured Comté (12 months) AOP	1248211	28/07/2022
21	Lidl	Netherlands	Cured Gouda	Cow	Jermikasewerk	Cured Gouda—Old Amsterdam (8 months)	2130753	25/07/2022
22	Mercadona	Netherlands	Aged Gouda	Cow	Vergeer	Aged Gouda	220614	10/11/2022
23	Lidl	Switzerland	Gruyere	Cow	Jermikasewerk	Gruyere Reserve (10 months)AOP	202 0702	17/07/2022
24	Mercadona	France	Gruyere	Cow	M&T	Gruyere AOP	7422154050545	07/10/2022
25	Mercadona	UK	Cheddar	Cow	Queseria La Fuente	Hacendado Cheddar	29042-166	13/09/2022
26	Mercadona	UK	Cured Cheddar	Cow	Dist. Juan Luna	Hacendado cured Cheddar	1072332	05/10/2022
27	Lidl	Italy	Parmigiano Regginano DOP	Cow	Consorzio Latterie Virgilio	Cured Deluxe Parmigiano (22 months)	22.119	04/09/2022
28	Family M	Netherlands	Maasdam	Cow	Dist. Juan Luna	Mature Maasdam	2237203	23/08/2022
29	Family M	Spain	Havarti	Cow	Lácteas Flor de Burgos S.L	La Bocateria Mature Havarti	28512137	14/09/2022
30	Family M	Netherlands	Edam	Cow	Dist. Juan Luna	Mature Edam	2237393	31/08/2022
31	Lidl	Germany	Emmental	Cow	Goldsteig Kasereien Bayerwald	Mature Milbona Emmental	192 M50	28/07/2022
32	Mercadona	Italy	Grana Padano	Cow	Zanetti S.p.A.	Cured Grana Padano AOP	L112236P	04.03.23

**Table 2:** Lactose and galactose concentrations obtained for the different samples analysed. Equivalent galactose concentrations were obtained by calculation from the sum of galactose and half the lactose concentration. AOP/DOP: protected designation of origin.

Nº	Brand Name	Lactose (mg/100 g)	Galactose (mg/100 g)	Galactose Equivalent (EG = ½ L + G) (mg/100 g)
1	Semi-cured Roncero	<5	40.4	<42.9
2	Semi-cured Hacendado	<5	83.5	<86
3	Semi-cured G.B.	<5	6.1	<8.6
4	Semi-cured Gran Capitán	<5	115.3	<117.8
5	Semi-cured Navidul	<5	32.7	<35.2
6	Cured Roncero	<5	509.8	<512.3
7	Cured Hacendado	<5	11.7	<14.2
8	Cured Gran Capitán	<5	283.7	<286.2
9	Gran Reserva Cured Lidl	<5	14.9	<17.4
10	Cured García Baquero Maestría	<5	33.3	<35.8
11	Cured Murcia al Vino DOP	<5	559.1	<561.6
12	Cured Mahón DOP	<5	<5	<7.5
13	Cured Idiazabal DOP	<5	120	<122.5
14	Aged García Baquero (10 months)	<5	41.5	<44
15	Aged Flor de Esgueva	<5	322.4	<324.9
16	Aged Hacendado	230	60	175
17	G.B. Reserva (12 months)	<5	12.3	14.8
18	Aged sheep’s Manchego DOP	<5	271.4	<273.9
19	Punteiro Tetilla DOP	11.8	13.9	19.8
20	Cured Comté DOP (12 months)	<5	8.6	<11.1
21	Cured Gouda—Old Amsterdam (8 months)	<5	5	<7.5
22	Aged Gouda	<5	5.5	<8
23	Gruyere AOP Reserva (10 months)	<5	<5	<7.5
24	Gruyere AOP	<5	<5	<7.5
25	Hacendado Cheddar	<5	285.5	<288
26	Hacendado cured Cheddar	<5	<5	<7.5
27	Cured Deluxe Parmigiano (22 months)	<5	<5	<7.5
28	Mature Maasdam	<5	<5	<7.5
29	La Bocateria Mature Havarti	<5	<5	<7.5
30	Mature Edam	<5	<5	<7.5
31	Mature Milbona Emmental	<5	<5	<7.5
32	Cured Grana Padano AOP	<5	<5	<7.5

**Table 3:** Lactose and galactose content of semi-cured cheeses of Spanish origin.

Name	Lactose (mg/100 g)	Galactose (mg/100 g)	Galactose Equivalent (GE = ½ L + G) (mg/100 g)
Semi-cured Roncero	<5	40.4	<42.9
Semi-cured Hacendado	<5	83.5	<86
Semi-cured García Baquero	<5	6.1	<8.6
Semi-cured Gran Capitán	<5	115.3	<117.8
Semi-cured Navidul	<5	32.7	<35.2

**Table 4:** Lactose and galactose content of cured cheeses of Spanish origin.

Name	Lactose (mg/100 g)	Galactose (mg/100 g)	Galactose Equivalent (GE = ½ L + G) (mg/100 g)
Cured Roncero	<5	509.8	<512.3
Cured Hacendado	<5	11.7	<14.2
Cured Gran Capitán	<5	283.7	<286.2
Cured Gran Reserva Lidl	<5	14.0	<17.4
Cured G.B. Maestría	<5	33.3	<35.8
Cured Murcia al Vino DOP	<5	559.1	<561.6
Cured Mahón DOP	<5	<5	<7.5
Cured Idiazabal DOP	<5	120	122.5

**Table 5:** Lactose and galactose content of mature and aged cheeses of Spanish origin.

Name	Lactose (mg/100 g)	Galactose (mg/100 g)	Galactose Equivalent (GE = ½ L + G) (mg/100 g)
Mature García Baquero (10 months)	<5	41.5	<44
Mature Flor de Esgueva	<5	322.4	<324.9
Mature Hacendado	200	27.5	127.5
García Baquero Reserve (12 months)	<5	12.3	14.8
Aged Sheep’s Manchego DOP	<5	271.4	<273.9

**Table 6:** Lactose and galactose content of cheeses of international origin.

Name	Lactose (mg/100 g)	Galactose (mg/100 g)	Galactose Equivalent (E = ½ L + G) (mg/100 g)
Cured Comté DOP (12 months)	<5	8.6	<11.1
Cured Gouda–Old Amsterdam (8 months)	<5	5	<7.5
Aged Gouda	<5	5.5	<8
Gruyere AOP Reserve (10 months)	<5	<5	<7.5
Gruyere AOP	<5	<5	<7.5
Hacendado Cheddar	<5	285.5	<288
Hacendado cured Cheddar	<5	<5	<7.5
Cured Deluxe Parmigiano (22 months)	<5	<5	<7.5
Mature Maasdam	<5	<5	<7.5
La Bocateria mature Havarti	<5	<5	<7.5
Mature Edam	<5	<5	<7.5
Mature Milbona Emmental	<5	<5	<7.5
Cured Grana Padano AOP	<5	<5	<7.5

## Data Availability

Not applicable.
